# Gasdermin E-mediated programmed cell death: An unpaved path to tumor suppression

**DOI:** 10.7150/jca.48989

**Published:** 2021-06-26

**Authors:** Yueyuan Wang, Jingyu Peng, Xiao Xie, Zhihao Zhang, Mingxi Li, Ming Yang

**Affiliations:** 1 Department of Breast Surgery, The First Hospital of Jilin University, Changchun, People's Republic of China.; 2 Department of Neurology and Neuroscience Center, The First Hospital of Jilin University, Changchun, People's Republic of China.

**Keywords:** GSDME, programmed cell death, cancer, immunity

## Abstract

Hearing loss-associated protein gasdermin E (GSDME), an effector of secondary necrosis, has been identified in a new pathway of programmed cell death (PCD). GSDME epigenetic silencing and mutations resulting in loss-of-function have been reported in cancer tissues. Additionally, GSDME upregulation inhibits tumor proliferation as well as colony forming ability, and reduces the incidence of lymphatic metastasis, demonstrating that GSDME may act as a tumor suppressor. Here, we have focused on the molecular mechanisms of GSDME-mediated PCD, and tried to reveal the crosstalk between this cell death pathway and apoptosis, autophagy, GSDMD-mediated pyroptosis. Moreover, we concluded the anti-cancer activity of GSDME include forming permeable membranes, and triggering anti-cancer immunity. Thus, GSDME was potential to be a novel target for cancer prevention and treatment.

## 1. Introduction

Gasdermin E (GSDME) belongs to the GSDM-family, which also includes GSDMA, GSDMB, GSDMC, GSDMD, and Pejvakin (PJVK) 1-3. GSDME has long been regarded to be associated with hearing loss (HL), in which its specific mutations form induces the occurrence of HL. The gene encoding GSDME is localized on human chromosome 7p15, and is also known as deafness autosomal dominant 5 (DFNA5) 4. It consists of 10 exons that encode a 496 amino acid protein with a molecular weight of 55 kDa 4-6. GSDME can be divided into three distinct domains, including a globular domain A (exon 2-exon 6), a hinge region (exon 6-exon 7), and a globular domain B (exon 7-exon 10) 6. As a member of gasdermin family which is widely expressed 2, the presence of GSDME gets a lot of attention. Till now, it has been found medium expression in brain, cardiac, and kidney cells, and low expression in cancer cells 7. The estrogen receptor in breast cancer cell lines is inversely associated with GSDME methylation and expression 8. Investigations into the relationship between GSDME and tumors have demonstrated that it is negatively related to proliferation, colony formation, invasion, and even the incidence of lymphatic metastasis 9, 10. Moreover, the level of GSDME expression has been linked to cancer patient outcome; a study showed that tumors deficient in GSDME developed drug resistance 5, and drug susceptibility was restored upon exogenous GSDME transfection in these cells 11.

Programmed cell death (PCD) pathways are crucial for growth, survival, homeostasis, and innate immunity of all multicellular organisms, and GSDME-dependent cell death is a newly discovered PCD. After GSDME was discovered to participate in cell death modulation 12, increasing attention was given to this gene. The GSDME-N domain shows necrotic activity, the toxic region is located in exons 2 and 6, and the GSDME-C domain (exon 8-10) regulates the necrosis-inducing activity, to prevent improper cell death 5, 6, 13-15. The N-terminal domain of GSDME mediates PCD with distinct morphological characteristics, affecting plasma membrane permeability and water influx, leading to cell swelling with typical bubbles and osmotic lysis 13, 14. Even though there are different forms of GSDME associated with HL on the genomic level, they all lead to exon 8 skipping with a complete structure of the necrosis-inducing part, which indicates that mutant type (MT) GSDME functions the same way as the GSDME-N-terminal domain 5. In addition, GSDME is one of the targets of p53, indicating that it may take part in cell cycle arrest and mediate PCD through the mitochondrial pathway 16-19.

In this review, we describe the molecular process of GSDME-mediated PCD and summarize therapeutic advances associated with GSDME and cancer suppression.

## 2. The crosstalk between GSDME-mediated PCD and conventional PCD

Resisting cell death leads to multiple diseases and is considered a hallmark of cancer 20. How to induce PCD in tumors is a popular research target in cancer therapy. Here, we listed several known PCD types, including apoptosis, pyroptosis (mediated by GSDMD) and autophagy.

Apoptosis is a process of cell death with cell shrinkage, which leads to a decrease in cell size and a denser cytoplasm. “Apoptotic bodies” consisting of cytoplasm with tight packed organelles are phagocytosed by macrophages or other cells, then degraded within phagolysosomes 21.

**Figure 1:**
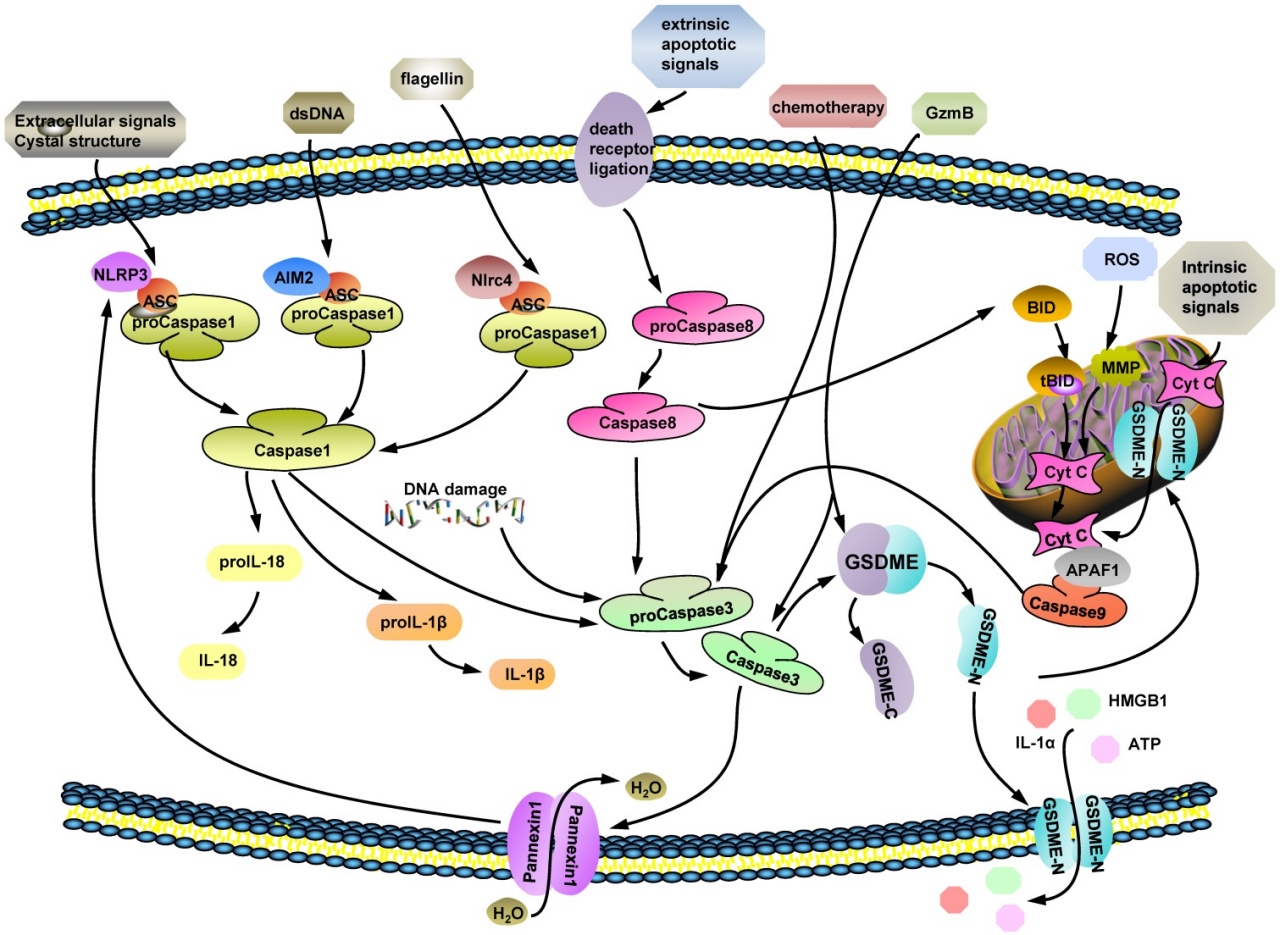
The possible molecular mechanisms of GSDME-mediated PCD. (ⅰ) Caspase 3 is a GSDME cleavage executor and facilitates to produce GSDME-N domain by cutting off full-length GSDME at D270. Ectocytic stimulus, such as: extracellular signals crystal structure, certain dsDNA and flagellin, mentioned in figure trigger caspase 1 activation by multifarious pathways. (ⅱ) Some extrinsic apoptotic signals are recognized by death receptor ligation on cytomembrane and active caspase 8. Both caspase 1 and 8 are upstream factors of caspase 3 and their activated forms induce caspase 3 cleavage. (ⅲ) Chemotherapeutic drugs and intracellular DNA damage stimulate procaspase 3 to turn into active caspase 3. (ⅳ) GzmB is a newly found factor which is effectively cut GSDME directly to release its N-terminal domain and takes roles in caspase 3 activation process as well. In conclusion, caspase 3 and GzmB, as the executors of GSDME cleavage, are modulated by various of cytokines.

Distinct with apoptosis, GSDMD-mediated pyroptosis is characterized by cells swelling, osmotic lysis and inflammasomes release 20. The important components of GSDMD-dependent pyroptosis are the inflammasomes, such as nucleotide-binding domain and leucine-rich repeat containing receptors (NLRs) and absent in melanoma (AIM)-like receptors 20, 22. Unlike AIMs activation, through sensing endogenous or pathogen-derived double-stranded DNA (dsDNA) directly 23-25, NLR (NLRP3, NLRC4) activators includes multiple pathogenic signals 25, 26, including reactive oxygen species (ROS), mitochondrial DAMPs, bacterial pore-forming toxins, extracellular ATP, uric acid crystals, which are sensed by NLRP3 27, 28, while flagellin and muramyl dipeptide are meant to active NLRC4 29, 30. Toll-like receptor 4 (TLR4) signaling triggers full inflammasome activation like NLRP3, by either post-translational modifications or subcellular re-localization of protein, while TLR4 co-engagement may also suppress inflammasome-dependent inflammation at later time-points 26. Pattern-recognition receptors (PRRs), recognize certain damage-associated molecular patterns (DAMPs) and pathogen-associated molecular patterns (PAMPs). Until then, they could assemble the apoptosis-associated speck-like protein containing a caspase recruitment domain (ASC), which links procaspase 1/8, to make up inflammasome complexes what are key to pyroptosis. ASC, as a bridge protein, binds to procaspase-1 through CARD-CARD interactions, and to procaspase 8, NLRs, and AIMs by pyrin domain (PYD)-PYD interactions, without interference 22, 31-34. The ASC assembly recruits procaspase-1, driving the activation of caspase-1 and -3 20, 35, 36. Then, GSDMD is cut by caspase 1 and divided into C-domain and N-domain which would bind to plasma membrane (Fig. 1).

Autophagy both mediates cell death and, under certain circumstances, helps cells to survive, which is the case during tumorigenesis and resistance 37-39. It has been illustrated that GSDME-N domain production elevation is related to protective autophagy attenuation, resulting in cell sensitivity to doxorubicin 40.

We collected the connections between these forms of PCD and GSDME-mediated PCD to provide insight into the current understanding of tumor resistance and cancer treatment development.

### 2.1. GSDME-mediated PCD and apoptosis

Apoptosis, a complex protease cascade process, is initiated by a wide-range of stimuli, including the MAPK-MEK pathway 41, oxidative stress 21, and intrinsic and extrinsic apoptotic signals. The most essential component of apoptosis is caspase-3, which also serves as the unique intrinsic signal that triggers GSDME cleavage. Additionally, apoptotic pathways are repressed in GSDME-knockout mice via gene expression microarray experiments 6. However, a number of genes are upregulated after MT GSDME transfection, and they modulate different apoptotic pathways, including oxidative stress, mitochondria dysfunction, pentose phosphate pathway activation, ER stress, and MAPK signaling 17-19, 42

MAPK pathways are continuously activated in yeast and human cell lines within exogenous MT GSDME, under mediation of extracellular signal regulated kinase (ERK), c-Jun N-terminal kinase (JNK), and p38, which is followed by involvement of certain mitochondrial proteins, including Fis1, Por1, Aac1, and Aac3 18. Furthermore, intrinsic and extrinsic apoptotic signals induce GSDME-N domain production 14, which in turn enhances apoptosis by permeabilizing the mitochondrial membrane and facilitating Cyt c release 43. Classic mitochondrial apoptotic pathways, such as the BIM-Bax-Cyt c-APAF1-Smac-caspase-GSDME axis, also play vital roles in GSDME-mediated PCD 11, 14. Overall, caspase 3-GSDME forms a self-amplifying feedback loop between GSDME-mediated PCD and apoptosis, as well as bridges extrinsic and intrinsic apoptosis pathways.

### 2.2. GSDME-mediated PCD and GSDMD-mediated pyroptosis

Programmed necrosis mainly refers to necroptosis, pyroptosis, ferroptosis, NETosis and mitochondrial permeability transition (MPT)-dependent necrosis 44. The cleavage of GSDME is induced by caspase 3 while GSDMD is triggered by caspase 1/4/5/11. Inflammatory caspases 1/4/5/11, initiator caspases 8/9, and the executioner caspase-7, are all unable to produce a pore-forming GSDME-N-terminal domain directly 13, 45. Therefore, GSDMD and GSDME were once thought to function independently. However, canonical inflammasomes (NLRP3, AIM2, and NLRC4), of which activation is the crucial step of GSDMD-dependent pyroptosis, function as early events leading to caspase-3 cleavage following caspase 1/8 activation 21, 35, 46, 47. Caspase-8 is not only the apical caspase in DNA-induced apoptosis, but induces caspase-3 activation in AIM2-, NLRP3-, and NLRC4-dependent pyroptotic pathways when caspase-1 is absent, with little or no requirement for either caspase-9 or caspase-2 46, 47. Additionally, caspase-1 facilitates caspase-3 activity regardless of whether caspase-8 is present 35. Therefore, caspase-3 cleavage is driven by caspase-1 or caspase-8. In addition, the channel-forming membrane protein pannexin-1 is a substrate of caspase-3/7 during apoptosis 47-49 and forms pores on plasma membranes, which allow for water influx but not inflammatory cytokines release 20.

ROS accumulation, DNA damage, and extrinsic and intrinsic apoptotic signaling induce caspase-3 activation 21, 47, 50. GSDME, downstream of caspase-3^13^, ^14^, ^45^, ^51^, can be cut into two segments; the GSDME-N-terminal domain that is capable of forming pores on cell membranes 13, 14, 43, 52, and the structural autoinhibition of gasdermin E is destroyed when some sites in GSDME-C terminal domain mutate 53 . The active form of GSDME permeates mitochondrial membranes and promotes cytochrome C (Cyt c) release and caspase-3 activation, contributing to a self-amplifying feed-forward loop, augmenting cytotoxic effects 43. Granzyme B (GzmB), released by natural killer (NK) cells, not only cleaves GSDME after D270, where the caspase-3 cleavage site was located, but cuts and activates caspase-3 54, 55. Overall, both caspase-3 and GzmB cleave GSDME effectively.

Cleaved caspase-3 blocks the GSDMD-mediated pathway specifically by cleaving GSDMD at D88 (D87 in human cells), deactivating the protein 14, 36. Overall, GSDME-mediated PCD and GSDMD-mediated pyroptotic pathways engage in bidirectional crosstalk in cell death processes (Fig. 1).

## 3. 3. GSDME and cancer

### 3.1. GSDME expression and tumorigenesis inhibition

Low level of GSDME expression is a common feature of most cancer cells. From epigenetic studies, GSDME epigenetic silencing occurs in about 52-65% of primary cancers 6, such as gastric cancer 56, colorectal cancer 57, 58 and breast cancer 9, 10; the incidence of GSDME hypermethylation indicated lymph node metastasis and a worse five-year survival rate in breast cancer 9, 10 as well as strong lymphatic vessel invasion and high TNM stage in colorectal cancer 58. At present, promoter DNA hypermethylation is the most common feature responsible for GSDME epigenetic inactivation 59. Moreover, some tumors avoid GSDME-mediated tumor suppression through loss-of-function mutations, which surround the caspase-3 cleavage site to abolish GSDME-mediated tumor cytotoxicity 54.

To identify whether GSDME is a tumor suppressor or not, a series of experiments were performed. Transfection of WT GSDME plasmids into cancer cells, including lung carcinoma, glioblastoma, melanoma, hepatocellular carcinoma, colorectal cancer, and triple-negative breast cancer cells, leads to significant tumor repression 6, 9, 43, 57, 60. Similarly, GSDME downregulation leads to an obvious increase in growth, colony formation and invasion, while G2/M arrest and caspase 3/8 activation by the Fas/FasL pathway after GSDME gene transfection 60, 61. When came to a mouse model of melanoma, GSDME-KO tumors behave faster than those expressing GSDME in not only formation but growing to reach sacrificial threshold 43. A GSDME-/- intestinal cancer mouse model showed a more atypical hyperplasia rather than tumor formation, and less moderate mucosal inflammation, than GSDME positive mice, in both chemically induced and genetically modified groups 62.

### 3.2. GSDME amplified PCD mechanism

#### 3.2.1. *In vitro*

Melanoma with GSDME deletion is known to resist etoposide treatment 9. The small molecular target drugs BRAFi + MEKi (the combination of BRAF inhibitors and MEK inhibitors), have been shown to kill tumors through MEK-ERK1/2 signal attenuation and mitochondrial depolarization, causing caspase-3 cleavage and GSDME-N domain accumulation. Cells resistant to this combination treatment do not undergo GSDME-mediated pyroptosis, unless treated with other drugs that stimulate the GSDME cleavage process 63. Additionally, iron synergizes with CCCP, which induces ROS signals, results in the mitochondrial outer membrane protein Tom20 oxidizing and oligomerizing. Bax, downstream of oxidized Tom20, is recruited to mitochondria to facilitate Cyt c release and activate the caspase cascade, ultimately leading to GSDME-N domain production 64. Additionally, protective autophagy, which usually occurs during chemotherapy, is a new challenge in melanoma treatment. Doxorubicin induces GSDME-mediated PCD, as well as eEF-2K activation, a kinase related to autophagy. Silencing eEF-2K amplifies GSDME cleavage triggered by doxorubicin, and decreases inappropriate autophagy, which leads to stronger tumor inhibition 40.

Polo-like kinase 1 (PLK1) inhibitor augments the chemosensitivity of esophageal squamous cell carcinoma cells through the BAX-caspase-3-GSDME pathway and inhibits DNA repair 65. In gastric cancer, intracytoplasmic contents flow from the pores formed by GSDME-N domain assembly upon 5-FU treatment 45. As_2_O_3_ exerts HCC suppression with GSDME cleavage and downregulation of DNMT-related proteins containing Dnmt3a, Dnmt3b, and Dnmt1, which take part in elevation of DNA methyltransferase expression 66, 67. In colon cancer cells, lobaplatin triggers ROS accumulation and JNK phosphorylation, resulting in intrinsic apoptotic signal initiation and GSDME-mediated PCD 14.

For lung cancer cells, whether traditional chemotherapeutic drugs or novel small molecular inhibitors, extrinsic and intrinsic apoptotic pathways are vital for induction of GSDME-N domain accumulation; the anti-tumor effectiveness of these drugs is weakened in GSDME deficient cells 11, 51. The mitochondrial pathway is prominent in the activation of GSDME 14, 18, 68. Dioscin induces a Bax/Bcl ratio decrease and GSDME-mediated PCD through the JNK/p38 pathway in human osteosarcoma 69. Osthole disequilibrates the mitochondrial membrane potential to raise ROS levels in ovarian carcinoma cells, resulting in GSDME-dependent pyroptosis rather than apoptosis 70. *In vitro*, galangin decreases glioblastoma multiforme cell, but not non-cancer cell, viability. It also triggers autophagy and GSDME-mediated PCD, where the Bax/caspase-3 axis plays an important role 71.

GSDME mRNA production in the human acute lymphoblastic leukemia cell line CEM increases after exposure to steroids. According to Webb et al., forskolin alone reduced cell growth but not death, however, it contributed to the apoptotic response when synergized with dexamethasone via the PKA cAMP pathway 72, 73. GSDME epigenetic inactivation is induced by promoter DNA hypermethylation commonly^59^, thus, decitabine, a common anti-cancer medicine, elevates tumor cell chemosensitivity through reversing GSDME silencing 56, 57

#### 3.2.2. *In vivo*

As the previous experiment mentioned, decitabine reversed GSDME silencing in human tumors 56, 57. This function was also confirmed in mice that decitabine significantly elevated the DFNA5 gene expression of macrophages, colon cancer cells, and breast carcinoma cells 74. It has been proved that treating specific tumor-bearing mice with combination of decitabine and cisplatin (DDP) in corresponding order, demethylating DFNA5 gene with decitabine primarily which followed by DDP, mediated a stronger tumor-killing effect than treating with DDP or decitabine alone. Moreover, a surprisingly increased presence of cytotoxic T lymphocytes (CTLs) in the tumor microenvironment was shown in mice treated with liposome cis-platinum + decitabine. This combination led to a shift of native CD8+T cells to the central memory CD8+T cells in the spleen, which considered to be efficient for mediating protective immunity 74. Experiment has been conducted recently to testify that ectopic mGSDME expression in 4T1E and B16 cells markedly inhibited tumor growth, whereas 4T1E tumors overexpressing lose-of-function (LOF) mutants of GSDME did not have more functional tumor infiltrating lymphocytes (TILs) 54. Moreover, regrowth of residual disease after removing BRAFi + MEKi was sooner and lager in GSDME-KO tumors than control group 63. Anyhow, the evidence to certify that GSDME serves as a cancer fighter by amplifying PCD is still far away from enough and fulfilled, lots of work called for been done.

#### 3.3. GSDME-mediated PCD activates the immune response

In 2011, Douglas Hanahan and Robert A. Weinberg extended the hallmarks of cancer from six to ten; avoiding immune destruction is one of the hallmarks 75. The pore-forming GSDME-N domain helps the release of pro-inflammatory factors (e.g. IL-1α, IL-1β, IL-18, high-mobility group box 1 (HMGB1) and adenosine 5′triphosphate (ATP)) in a lysis-dependent manner 13, 14, 43, 55, 63, 76, leading to a strong inflammatory reaction even though it has nothing to do with IL-1β/18 maturation. Immunocytes receive these stimuli, like HMGB1 and ATP, and progress to cell death processes. Additionally, its expression relates to the tumor microenvironment, which stimulates the recruitment of tumor infiltrating lymphocytes (TILs), tumor-associated macrophages, and inflammatory signal production, like GzmB and perforin (PFN) 54. Due to immunity participation, GSDME-mediated anti-cancer pathways involve systemic responses and does not kill tumors alone.

#### 3.3.1. Phagocytosis amplification and immunocytes activation

Pro-necrotic molecules promote intra-tumoral immunity through immunocytes, thus, GSDME, a pro-necrotic molecule, is significantly positively associated with myeloid cells, especially macrophages 77. Consistently, the markers of macrophages increase significantly in tumors with ectopic GSDME expression 54, indicating that GSDME enhances the phagocytosis of tumors. The activation and proliferation of TILs is beneficial in sustained tumor suppression 78. NK cells in ectopic GSDME expression tumors express more GzmB and PFN, similar to CD8+ T cells, although with no increase in quantity 54. Of note, there is more secretion of IFNγ and TNF from CD8+T cells, suggesting the elevation of immunocyte activation. Furthermore, BRAFi + MEKi induces GSDME-mediated PCD, following by intra-tumoral immune reactions involving the release of inflammatory factors, intra-tumor dendritic cell (DC) activation, and T cell abundance extension. Less immunocyte activation and shorter tumor regression under BRAFi+MEKi therapy are detected in GSDME-knockout tumors than in control counterparts 63. This gene is well-correlated to the anti-tumor immunocyte response.

#### 3.3.2. GzmB from NK cells

GzmB, an exogenous serine endopeptidase, is found in a variety of cancers. It is generally released from TILs with PFN, and regulates caspase-independent cell death upon intracellular delivery of PFN 79, 80. When GSDME-overexpressing tumors are incubated with NK cells, GzmB, from NK cells, cleaves GSDME effectively and initiates GSDME-mediated pyroptotic pathway 54. Similarly, during chimeric antigen receptor (CAR) T cell therapy, cytolytic T cells release GzmB and drive GSDME cleavage. This reaction triggers cytokine release syndrome (CRS) in patients, neutralizing the effectiveness of CAR T treatment. Furthermore, the effect of GzmB is amplified by caspase, for it cuts caspase-3/7 to produce their active forms 55, 79, 80. Taken together, GzmB mediates extrinsic and caspase-independent pathways of GSDME-mediated PCD.

## 4. Conclusion

While GSDME has been of interest for decades, associated studies remain limited. GSDME has been shown to play a crucial role in various molecular processes, including HL 4, 81, tumor suppression 57, PCD-mediation 6, 18 and acting as an anti-tumor immunity promoter 54, 63. However, recent research found out that treating GSDME-/- mice with cisplatin, they showed less tissue damages and weight loss 13. Therefore, GSDME was benefit for anti-cancer therapy outcomes or not was still unclear and more experiments combining GSDME and anti-cancer therapy was urged to conducted.

Despite adverse reactions, such as CRS 55, severe weight loss upon chemotherapy 13, and induction of renal tubulointerstitial fibrosis progression 82, the significant anti-tumor potential of GSDME should not be ignored. Some researchers propose that GSDME may be a promising biomarker for cancer detection, for predicting the 5-year patient survival rate 83, and for use in GSDME-mediated PCD initiated immunogenic cell death (ICD) in GSDME-overexpressing cells 54. The standard of ICD is that secondary-tumor formation could be prevented after injection with tumor cells experiencing ICD 84, meaning that GSDME-dependent cell death results in sustainable tumor regression. P53, an acknowledged tumor suppressor, commands various of downstream genes transcriptional activation. DFNA5, one of those genes, has been performed to be strongly induced by exogenous and endogenous p53. Concertedly, the ability of promoting cell death of DFNA5 can only be exerted in the presence of p53 while its effect reversed in the absence of p53 to entice the resistance to etoposide. A possible explanation of this relation is that a potential p53-binding sequence locates in DFNA5 intron 1 and DFNA5 is a mediator in p53-inducing cell death process, thus DFNA5's function might be disordered when p53 is negative or LOF 16.

More than acting as a tumor suppressor, GSDME functions as a potential therapeutic target in the battle against cancer. It has been proved that chemotherapeutic drugs like cisplatin, paclitaxel or doxorubicin successfully induced GSDME-N domain formation, which was benefit to cancer cells killing, including lung cancer 51 and colon cancer 85. Small molecular inhibitors, effecting on KRAS, EGFR, ALK in lung cancer 11, BRAF and MEK in melanoma cells 63, elicit concurrent GSDME-mediated PCD. Additionally, PLK1 kinase inhibitor can be utilized as adjuvant in cisplatin-driven oesophageal squamous cell carcinoma therapy since it enhances the chemosensitivity by eliciting GSDME cleavage 65. Excessive levels of ROS increase cellular oxidative stress which is harmful for cell surviving and disruption of ferritin lead to elevation of ROS 64. Iron combining with ROS-inducing drugs facilitates ROS level to be sensed by Tom20-Bax-caspase-GSDME axis with mitochondrial intrinsic apoptotic dependency, what has been detected by Zhou's group. Interestingly, knockdown GSDME could attenuate pyroptosis and LDH release while this inhibitory effect is abolished when GSDMD replaced GSDME 64. Hence, GSDME might serve as an executor in cancer cells death. But, the detail of how GSDME mediates cancer therapy has not been completely revealed and whether GSDME cleavage boosts cancer treatment *in vivo* has not been ultimately confirmed.

Currently, the clinical application of GSDME remains a future prospect, and the molecular mechanisms underlying its activity remain to be precisely characterized. Further studies that unveil GSDME biological functions will improve the current understanding of its role in cancer proliferation and invasion, which will expedite the development of novel anti-cancer therapies.

## Abbreviations

GSDME: gasdermin E
PCD: programmed cell death
PJVK: Pejvakin
DFNA5: deafness autosomal dominant 5
ICERE-1: inversely correlated to estrogen receptor expression
MT: mutant type
PRR: pattern-recognition receptor
DAMPs: damaged-associated molecular patterns
PAMPs: pathogen-associated molecular patterns
NLRs: nucleotide-binding domain and leucine-rich repeat containing receptors
AIM: absent in melanoma
dsDNA: double-stranded DNA
ROS: reactive oxygen species
ASC: apoptosis-associated speck-like protein containing a caspase recruitment domain
CARD: N-terminal caspase recruitment
PYD: pyrin domain
Cyt c: cytochrome C
GzmB: Granzyme B
NK: natural killer
ERK: extracellular signal regulated kinase
JNK: c-Jun N-terminal kinase
eEF-2k: Eukaryotic elongation factor-2 kinase
MAPK: mitogen-activated protein kinase
BRAFi + MEKi: the combination of BRAF inhibitors and MEK inhibitors
CCCP: carbonyl cyanide m-chlorophenyl hydrazine
PLK1: Polo-like kinase 1
DNMT: DNA methyltransferase
PKA: protein kinase A
cAMP: cyclic adenosine moriophosphate
TILs: tumor infiltrating lymphocytes
IL-18: Interleukin 18
IL-1β: Interleukin1β
PFN: perforin
IFNγ: interferon γ
TNF: tumor necrosis factor
DC: dendritic cell
CAR: chimeric antigen receptor
CRS: cytokine release syndrome
ICD: immunogenic cell death
TNM: tumor, lymph node, metastasis
HMGB1: high-mobility group box 1
ATP: adenosine 5′triphosphate
CTLs: cytotoxic T lymphocytes
DDP: cisplatin
TLR: toll-like receptor
LOF: lose of function
